# Gut microbiota and immune crosstalk in metabolic disease

**DOI:** 10.1016/j.molmet.2016.05.016

**Published:** 2016-06-06

**Authors:** Rémy Burcelin

**Affiliations:** 1 Institut National de la Santé et de la Recherche Médicale (INSERM), Toulouse, France; 2 Université Paul Sabatier (UPS), Unité Mixte de Recherche (UMR) 1048, Institut des Maladies Métaboliques et Cardiovasculaires (I2MC), F-31432 Toulouse Cedex 4, France

**Keywords:** Type 2 diabetes, Obesity, Microbiota, Intestinal immune system, Bacterial translocation, APC, antigen presenting cells, ILC, innate lymphoid cells, AMP, anti-microbial peptides

## Abstract

**Background:**

Gut microbiota is considered as a major regulator of metabolic disease. This reconciles the notion of metabolic inflammation and the epidemic development of the disease. In addition to evidence showing that a specific gut microbiota characterizes patients with obesity, type 2 diabetes, and hepatic steatosis, the mechanisms causal to the disease could be related to the translocation of microbiota from the gut to the tissues, inducing inflammation. The mechanisms regulating such a process are based on the crosstalk between the gut microbiota and the host immune system. The hologenome theory of evolution supports this concept and implies that therapeutic strategies aiming to control glycemia should take into account both the gut microbiota and the host immune system.

**Scope of review:**

This review discusses the latest evidence regarding the bidirectional impact of the gut microbiota on host immune system crosstalk for the control of metabolic disease, hyperglycemia, and obesity. To avoid redundancies with the literature, we will focus our attention on the intestinal immune system, identifying evidence for the generation of novel therapeutic strategies, which could be based on the control of the translocation of gut bacteria to tissues. Such novel strategies should hamper the role played by gut microbiota dysbiosis on the development of metabolic inflammation.

**Major conclusions:**

Recent evidence in rodents allows us to conclude that an impaired intestinal immune system characterizes and could be causal in the development of metabolic disease. The fine understanding of the molecular mechanisms should allow for the development of a first line of treatment for metabolic disease and its co-morbidities.

This article is part of a special issue on microbiota.

## 1 Introduction

**Figure 1:**
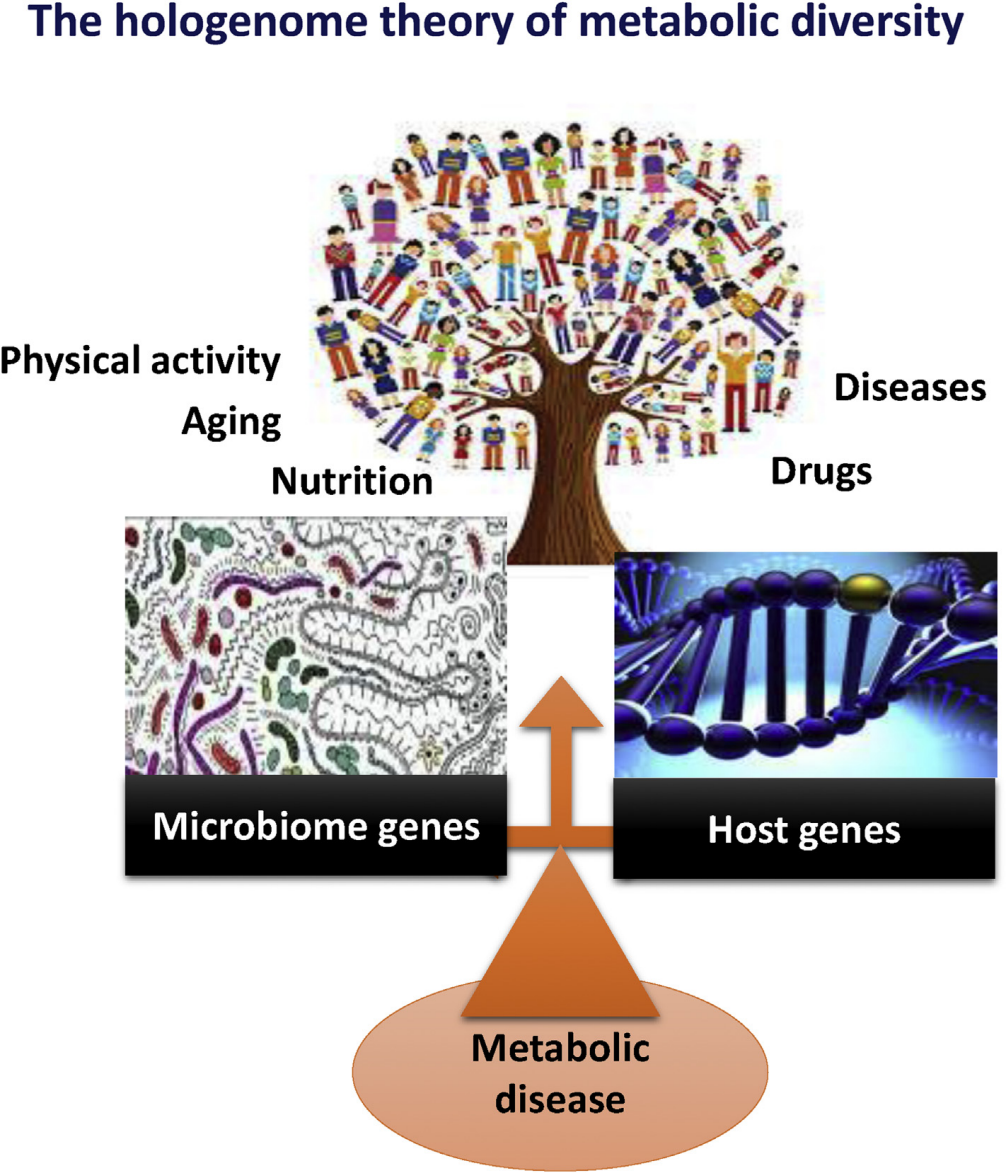
**The hologenome theory of metabolic diversity**. Gut microbiota metagenomics diversity and the host genetic diversity regulate human metabolic diversity. This balance is under the control of aging, food, drugs, physical exercise, and diseases to cite a few.

The hologenome theory of evolution proposes that natural selection acts not on the individual organism but on the “holobiont”, which consists of the host organism together with its microbiome (its genes and metabolites). When a holobiont is challenged by dramatic changes, such as altered diet, reduced physical activity, aging, drugs, or a disease, it employs adaptive mechanisms in the form of reshuffling/balancing its microbiome, i.e. resident microbial communities (Figure 1). The host side of the holobiont should also evolve and adapt to the changes. The mechanisms of this binary evolution, i.e. molecular crosstalk, still remain to be precisely determined, although evidences are on the way.

Within the holobiont, the most intuitive counterpart to the microbiome, which can be considered as the best-fit candidate to the microbiome diversity and the hologenome theory of evolution, is the immune system. The swiftness of its adaptability and the plasticity of the genome of the immune cells are such that changes in microbiota can be detected within days or weeks, allowing an adapted response of the host. Consequently, a major dysregulation of one of the components is likely to dramatically impact the other and, therefore, the holobiont. However, Darwinian selection, inherent to the hologenome theory of evolution, allows a drastic elimination of all dysregulated relationships between the microbiome and the immune system, which are at risk to the health of the holobiont. Therefore, to explain the epidemic development of metabolic disease, it is necessary to understand the molecular mechanisms responsible for imbalances combining subtle or mild impairments to the microbiome and host adaptability to environmental or evolutionary conditions. To design treatments for chronic, metabolic impairments, one should consider the molecular underpinnings of both the impaired microbiome and the impaired immune system

The causal role of gut microbiota on metabolic diseases has been shown in rodents through microbiota transfer experiments [1] and in humans [2] demonstrating that the microbiota from a healthy donor could improve the body weight and glycemia of an obese and diabetic receiver, respectively. Specific mechanisms of the host adapting to the change in microbiota could be proposed but would not fit with the notion of holobiont adaptation. Conversely, the immune system is the first line of adaptation to changes in the microbiome, where the innate immune system is, in a broad and non-specific manner, the fastest to adapt. It is followed by the adaptive immune system, which provides specificity, swiftness, and a memory to the dysbiotic microbiome. This crosstalk could be the first concept integrating the impact of the environment (social, nutritional, chemical, and behavioral) with the genetic of the host to explain the diversity and the development of metabolic disease in light of the definition of the holobiont.

This review will highlight the recent discoveries in the microbiota to host immune crosstalk with respect to metabolic disease. It will also aim to promote the concept that the development and treatment of metabolic disease should take into consideration that the holobiont, including the microbiome and immune system as the master regulatory mechanism, is a complex organism adapted to the environment. A change of the environment could impact both the host and the microbiome, leading to the development of metabolic disease. The role of the immune system as a key adaptor to the impact of the environment on the microbiome will be developed within the framework of metabolic disease.

It could be thought that at the cutting edge of the buffering capacity of the host and of the microbiota to adapt to the environment would be a thin line separating homeostasis and pathology. One should consider, however, that homeostasis and pathology are on a continuum, resulting from the adaptive capacity of the microbiome ecology and of the host, i.e. the immune system, to adapt to the environment. The threshold of a given molecular mechanism of the microbiome to host crosstalk, classifying individuals in a state of health or of metabolic disease, should always be considered for a specific homogeneous group of individuals in a specific environment. Classifying biomarkers and tailoring medicine/nutrition strategies should be based on an understanding of the adaptation of the holobiont – the host, its immune system, and its microbiome.

## 2 Metabolic disease: the “infectious” origin and the role of bacterial translocation

**Figure 2:**
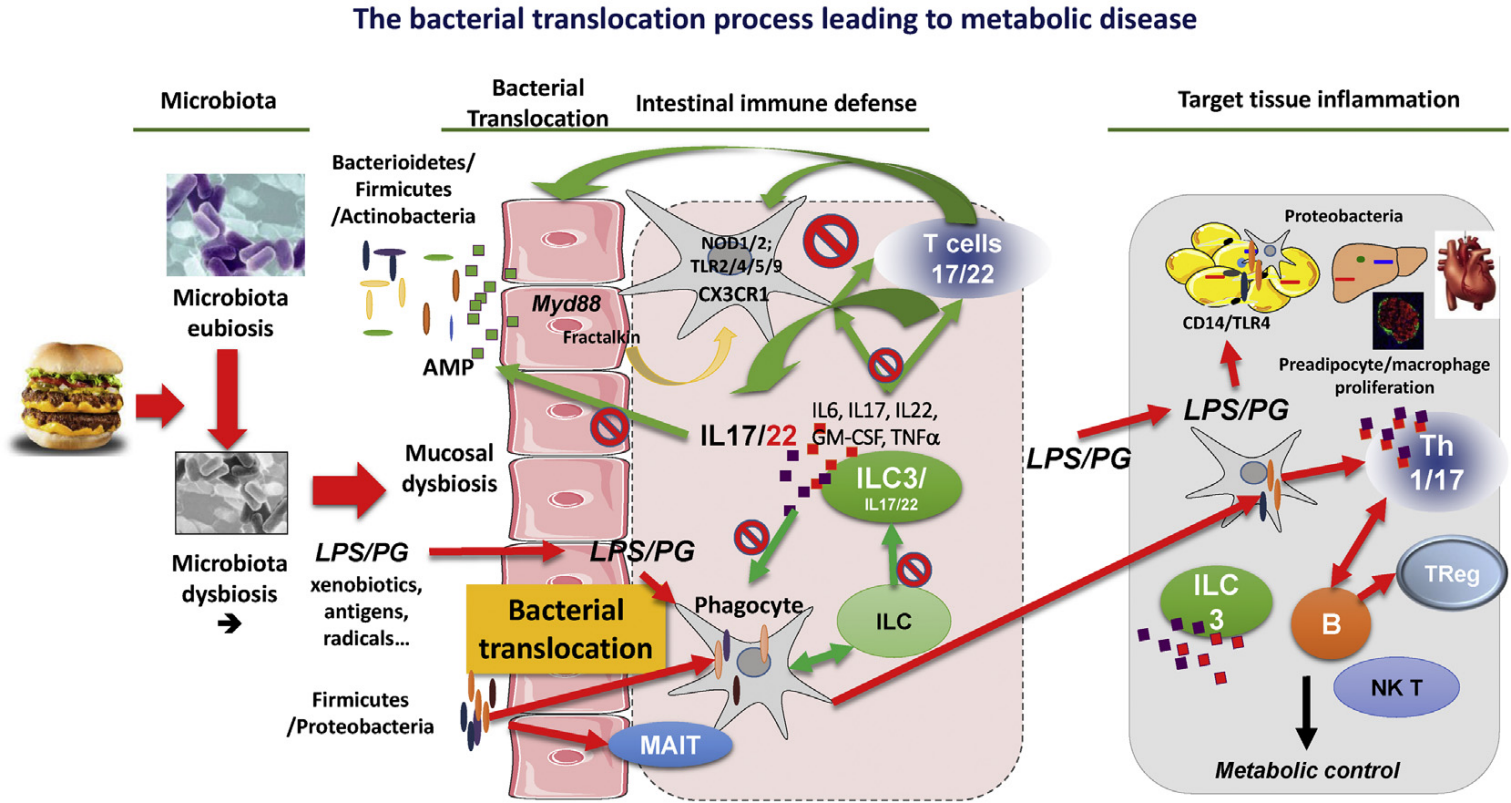
**Gut microbiota to intestinal immune defense interplay for the control of bacterial translocation-induced tissue inflammation and metabolic control**. A eubiotic gut microbiota is composed of a diverse bacterial community, which may reside within the intestinal mucosal layer but predominantly reside in the lumen. AMP or defensin and immune cells prevent from the mucosal adherence and translocation of the luminal bacteria (as shown by the green arrows). Upon a gut microbiota dysbiosis, such as induced by a fat-enriched diet, a dysbiotic mucosal microbiota composed of Proteobacteria and Firmicutes appears, impairing intestinal epithelial cell function and AMP production and leading to increased gut permeability. During the development of metabolic disease (depicted by the red arrows and tags), the mucosal bacteria and corresponding fragments such as the LPS and peptidoglycan translocate through the epithelial layer reaching the *lamina propria* where the phagocytes capture the bacteria. Gut microbiota dysbiosis impairs the crosstalk between the phagocytes, the ILCs, and the T cells. The co-activation between phagocytes and T cells is notably impaired, reducing Il22/17 production. An impaired ILC and MAIT cell function could be expected but requires more studies. Altogether, the impaired adaptive and innate immune defenses allow for the translocation of bacteria and bacterial components LPS and peptidoglycan towards metabolic tissues such as adipose depots, the liver, the islets of Langerhans, or the heart/vessels. On site, they trigger inflammation leading to proliferation of preadipocytes and macrophages so that the corresponding cytokines contribute to a reduced insulin signaling. A compartmentalization mechanism occurs since ILC3 frequency increases in the tissues, further increasing inflammation through the release of cytokines. The tissues are also characterized by increased infiltration of B and T lymphocytes, which interact with newly infiltrated phagocytes and further aggravate inflammation.

Metabolic diseases, obesity, and type 2 diabetes are multifactorial, chronic, non-communicable diseases for which the past decades of research have aimed at identifying a genetic origin. After extensive analyses, no more than 2–3% of the incidence of metabolic diseases can be explained by more than 30 gene loci when combined together [3]. This resides in the fact that a novel paradigm engulfing numerous causal mechanisms is needed to uncover the epidemic development of the disease. During the last decade, it was identified that the vast majority of obese rodents and patients are characterized with a gut microbiota dysbiosis [4], [5], [6], [7] (Figure 2). However, several bacterial entities, as defined by enterotypes, have been identified [8], highlighting the notion that not all obese patients are characterized with similar gut microbiota dysbiosis [7] and underlining several gut microbiota dysbiosis-dependent mechanisms. The gut microbiota encompasses more than 10 million genes [9] while eukaryotic cells, on average, express 25 thousand genes. This genomic potential sets up the basis of a complex holobiont, which should be well adapted to environmental changes. For the first time, the crosstalk between the large metagenomics and genomic diversities could provide an explanation of the pandemic development of metabolic diseases. However, the drawback of this phenomenon resides in genomic complexity. To decipher the corresponding molecular mechanisms, non-a priori multiOmics strategies from large cohorts and relevant animal models are required to validate the hypotheses. Through tremendous efforts the scientific community has identified that Firmicutes and Bacteroidetes are the two major phyla constituting fecal microbiota, with Actinobacteria contributing less than 5%, although the role of this is not negligible [10]. From that taxonomic and bacterial gene catalog, signatures classifying patients according to their disease, such as obesity and type 2 diabetes, have been identified [11]. Among them is the reduced proportion of some universal butyrate-producing bacteria as well as an enrichment of other microbial functions conferring sulphate reduction and oxidative stress resistance. It is noteworthy that the gene catalog has been established from fecal samples and that, throughout the digestive tract (starting from the mouth), pH, nutrient, oxygen, and bile acid gradients are major factors shaping the bacterial ecology, which cannot be identified from fecal sequencing. This is of major importance when one considers understanding the crosstalk between intestinal microbiota and the host since the signature of the gut microbiota dysbiosis along the digestive tract remains to be established in human. Ever since, numerous studies have been undertaken to identify dysbiotic fecal microbiota signatures of disease or of the environmental influence. The first evidences came from different diet-fed mice [12] and in humans treated with prebiotics [13], [14]. A major impact is observed in mice fed a high-fat diet [5], [15], [16], [17] in which the mechanisms involved in the development of the metabolic disease were related to the translocation of bacterial components such as lipopolysaccharides [5] and full bacteria [18] (Figure 2). In rodents, it is notable that a proliferation of Proteobacteria was observed at the mucosal layer (Figure 2) [19], [20], leading to a local increase of lipopolysaccharides [21].

The first proof of concept regarding the causal role of gut microbiota in the control of body weight gain was provided in 2004 when it was shown that germ free mice fed a high-fat diet gain less weight than conventional mice despite increased food intake [22]. The mechanisms for this difference were associated with an increase of the non-insulin dependent AMP-activated kinase pathway. AMPK controls energy expenditure by increasing glucose oxidation in situation of metabolic stress such as hypoxia fasting and exercise. In a related experiment, gut microbiota transfer was performed between obese conventional and lean germ free mice. This experiment demonstrated the causality of gut microbiota in obesity since germ free mice gained more weight when colonized with the microbiota from obese than from lean mice [23]. The authors of this study identified the mechanism as increased energy efficiency with the potential for the gut microbiota to harvest more energy from the diet. Human studies of fecal transplant showed a small but significant improvement of the glycemic control and insulin action, as assessed by the hyperinsulinemic clamps, when the microbiota from a healthy donor was transplanted to a type 2 dependent patient [2]. The improved insulin action in a subset of patients was maintained over 3 months and was dependent upon the transplant recipient, suggesting that a mechanism from the host is most likely a major regulator of the efficacy of the graft process. Unfortunately, the studies could not identify the host dependent mechanism responsible for the graft efficiency.

In the last decade, other mechanisms reconciling the immune system, particularly as relates to metabolic inflammation, with gut microbiota dysbiosis have been proposed. Metabolic diseases are characterized by a state of a progressive development of a low-grade inflammation in metabolic tissues [24], [25], [26] such as adipose, liver, muscles, and pancreatic islets. Innate immune cells infiltrate the tissues through a mechanism requiring the expression of the C–C motif chemokine receptor-2 (CCR2) by the circulating monocytes tissue macrophages and the production of chemokine ligand-2 (CCL2) and MCP1 by adipose tissue derived cells [27]. Activated M1 macrophages produce large amounts of TNFα, IL-1β, and IL-6, which contribute to insulin resistance by phosphorylating the c-Jun amino terminal kinase (JNK) and inhibitor of nuclear factor kappa B kinase subunit β (IKK-β) responsible for the phosphorylation of serine of IRS-1 [28], [29]. Serine phosphorylation inactivates the IRS, which reduces insulin signaling and triggers insulin resistance. Conversely, in lean animal models, the alternatively activated M2 macrophages produce mainly anti-inflammatory cytokines, such as IL-10, that maintain insulin sensitivity [30]. The balance between M1 and M2 is under the control of T and B cells through the production of the cytokines IFNγ and TNFα that favor M1 or through the production of IL4-5-10-13, which favors M2. Recent evidence demonstrates that the adipose tissue of high-fat diet-fed mice is infiltrated by adaptive immune cells, among which are T lymphocytes [31], [32], [33], [34] and natural killer T cells [35]. The causal role of the latter was demonstrated when the mice were treated with alpha-galactosylceramide, which activates the NK T cells and induces adipose tissue macrophages infiltration, inflammation, and insulin resistance [35]. The infiltration of the T lymphocytes was most likely specific since the adipose tissue CD4-T cells were characterized by biased T cell B receptor V-alpha repertoires, suggesting an antigen-specific expansion [31], [32], [33]. CD4-T cells reversed weight gain and insulin resistance both by their transfer into lymphocyte-free Rag1-null DIO mice as well as through the treatment with CD3-specific antibody or its Fab fragment, demonstrating a causal role [31], [32]. In other instances, it was observed that CD8-T cells infiltrate the adipose tissue of obese mice, which precedes the accumulation of macrophages [33]. Causality was demonstrated since the genetic depletion of CD8-T cells lowered macrophage infiltration and adipose tissue inflammation and ameliorated systemic insulin resistance [33]. Conversely, adoptive transfer of CD8-T cells to CD8-deficient mice aggravated adipose inflammation.

The role of B-cells in obesity-related immunometabolism should also be considered since these cells infiltrate the adipose depots [31]. Recent evidence shows that immune cells are compartmentalized by body tissues during obesity, notably in response to a high-fat diet [34]. Converging evidence from different groups has shown that the frequency of IL-17 and IL-21/22 producing cells and other related Th17 responses increased in liver and adipose depots while being dramatically reduced in the gut [34], [36]. B cells also contribute to metabolic inflammation, and ultimately insulin resistance, by presenting antigens to T cells, leading to the production of inflammatory cytokines as well as pathogenic IgG antibodies [31]. B cells reduce the number of regulatory T-cells and, overall, control T-cells activation [37].

Altogether, the infiltration of adaptive immune cells, characterized by a specific repertoire within the stroma vascular fraction of metabolic tissues during diet-induced obesity, seems to be a first step towards metabolic inflammation. This suggests that a specific antigen within the metabolic tissues is responsible for the lymphocyte repertoire. The secondary infiltration of adaptive immune cells leading to the production on site of inflammatory cytokines interleukin (IL) 1b, and TNFα, and interferon γ creates a vicious cycle. Yet unknown antigens accumulate in tissues encountering resident innate immune cells [38], which could educate adaptive immune cells, leading to the production of chemokines. Specific chemokine-sensitive monocytes and macrophages would be attracted by the chemokines from the tissues and further reinforce inflammation in the tissues. A key question resides in the identification of the antigens accumulating within the tissues.

Recent evidences from our laboratory suggested that bacterial fragments from the gut microbiota could translocate to tissues and initiate metabolic inflammation [39]. We observed that lipopolysaccharides (LPS) from intestinal gram negative bacteria accumulate in the blood of HFD-fed mice establishing a state of metabolic endotoxemia [5]. It is noteworthy that the endotoxemic profiles followed the nictemeral cycle, in which the concentration of plasma LPS was the highest at the end of the feeding period and lowest during the resting period, corresponds to changes in the gut proliferation of the bacteria following feeding. We then validated that apparently healthy individuals fed a fat-enriched diet rather than on a carbohydrate and protein-enriched diet were characterized by higher concentration of blood LPS [40]; individuals with type 2 diabetes and dyslipidemia also have higher concentrations of LPS [41]. The first causal role of metabolic endotoxemia in the development of metabolic disease was shown when a chronic, one month long, low grade infusion of LPS was performed in the mouse [5]. Hepatic insulin resistance, hyperglycemia, and reduced glucose-induced insulin secretion were induced by the LPS infusion. Furthermore, body weight gain and adipose tissue precursor proliferation were directly dependent upon the triggering of a CD14-dependent mechanism [42] associated with the local tissue activation of macrophage proliferation. This observation was then demonstrated *in vivo* through an intravenous bolus of lipopolysaccharides (4 ng/kg), which caused an early and constant increase in interstitial pyruvate, serum lactate levels, and glycerol 90 min after LPS treatment, demonstrating the metabolic impact of LPS in humans. The endotoxins can be absorbed during the synthesis of chylomicrons by the gut [43] and are the most bacteria-produced inflammatory molecules, requiring more than 20 genes to be produced [44]. Furthermore, according to its hexameric or pentameric structure, LPS could trigger or inhibit TLR4/CD14-induced cytokine production, respectively [45]. The inflammatory activity of LPS molecules can be toned down by the circulating soluble CD14 [46], [47], [48], [49] and by LPS binding proteins [50], which are secreted by the immune cells [47] and the adipocytes [51], [52] controlling consequently inflammation and insulin resistance [53], [54]. LPS can also be transported by lipoproteins to the adipose cells [55], buffering their proinflammatory activity. However, LPS can also oxidize lipoproteins and induce the production of superoxide anion by endothelial cells, further enhancing the inflammatory process [56]. Consequently, LPS can be released from the oxidized lipoprotein within metabolic organs to further induce inflammation [57], [58]. A process involving the sCD14 and lipoprotein lipase to liberate the free fatty acids [57], [59] as well as apoprotein exchange between lipoproteins could be considered regulating inflammation [60], [61], [62]. The chylomicrons can be used to buffer LPS and reduce inflammation [63], [64] as demonstrated by the activation of NFkB on rat hepatocytes. We observed in type 2 diabetic patients that plasma LPS levels were not different from those in controls, but LPS distribution in the two groups was different [65]. Patients with T2DM had higher levels of LPS-VLDL and free LPS free (non-lipoprotein bound) and lower levels of LPS-LDL, demonstrating a dysregulation in the LPS exchange between lipoproteins. In humans, free LPS transfers first to HDL and then to VLDL, whereas the LPS-bound LDL fraction is mainly derived from VLDL catabolism, which could represent a LPS catabolic pathway impaired in T2DM patients leading to inflammation. Altogether, the homeostasis of LPS and its role in the induction of inflammation is subtle and, undoubtedly, under the control of numerous factors. A naïve plasma LPS assay cannot be considered as pro or anti-inflammatory if one does not consider the LPS-binding proteins and the cellular environment.

Impaired gut permeability, therefore, is responsible for increased bacterial translocation. Several mechanisms can trigger acute bacterial translocation, as demonstrated following simple irradiation studies [66]. Consequently, the simple translocation of bacteria or bacterial compounds such as peptidoglycans/LPS could trigger the innate immune system. In a homeostatic situation, the corresponding inflammatory reaction in tissues can increase the vigilance, favoring defense mechanisms to fight against tumor cell proliferation [66]. However, in non-homeostatic conditions such as during metabolic disease, the shifted paradigm towards a deleterious mechanism results in chronic, impaired gut permeability, which leads to a continuous translocation of bacteria and bacterial factors towards tissues, ultimately triggering a long term inflammatory process. This chronic, inflammation could become deleterious for insulin action and secretion, i.e. diabetes and obesity [67].

As discussed above, high-fat diet induces impaired gut permeability [68] through mechanisms impairing the expression of genes that the proteins ensure a tight junction of epithelial cells [6]. Occludins are a group of proteins that the expression and the phosphorylation in the intestine is impaired and could be responsible for the paracellular translocation of bacterial components [69]. Insulin regulates the phosphorylation of proteins from the cytoskeleton through the activation of the myosin light chain kinase favoring leakiness of the gut during and after a meal [70]. Hence, bacterial fragments could physiologically translocate from the gut to systemic circulation to tissues. Therefore, the quality and the quantity of the bacterial molecules translocated could depend upon the mucosal gut microbiota composition. A dysbiotic gut microbiota could lead to the translocation of bacteria and bacterial fragments considered differently by the host. Importantly, through the impaired tight junctions, dendritic cells can sample mucosal bacteria and translocate the live intracellular bacteria to tissues [71]. The translocation of full live bacteria from the intestine to tissues has been described in many instances [72] such as in response to a state of immunodeficiency [73], major tissue stress [74], short bowel inflammatory disease [75], infections [76], and streptozotocin-induced Langerhans islet cell destruction [77]. We demonstrated in HFD-fed mice that GFP-labeled bacteria, when gavaged to a diabetic, obese mouse, can reach the adipose depot within two hours following the administration [18] (Figure 2). Since they are engineered to be resistant to ampicillin, colony forming units were observed growing on plates containing ampicillin, demonstrating that live bacteria from the gut reached the tissue [18]. This process was mediated by immune cells expressing Nucleotide-binding Oligomerization Domain receptor (NOD) 1 since the deletion of the corresponding gene prevented the translocation of bacteria and the development of metabolic disease. Furthermore, in other instances, immunosuppressive agents such as cyclophosphamide inhibited the translocation from the gastrointestinal tract to the mesenteric lymph nodes and reduced the numbers of lymphoid cells, especially B cells, in the Peyer Patches, the mesenteric lymph nodes, and the spleen, suggesting a role for these cells in the translocation process [78].

The physiological or deleterious importance of the bacterial translocation process in the development of metabolic disease remains to be determined. Evidence demonstrates that the beneficial effect of bacterial translocation is related to the development of the intestinal immune response by physiological interaction between bacteria and host [76], [79]. The beneficial effect involves degradation of the intestinal mucosa layer by bacteria such as probiotics [80], which enhance the intestinal defense of the host against putative further invasion leading to the translocation of deleterious bacteria towards tissues. The mechanisms of bacterial translocation involve the identification of the bacteria by the host through microbe associated pattern receptors such as the NOD [81]. In HFD-induced metabolic disease we demonstrated the protective role of NOD2 in the control of glycemia and insulin resistance [18], [82] and the deleterious role of NOD1 in favoring the translocation of bacteria [18]. When binding NOD2 expressed by innate immune cells, specific bacterial peptidoglycan residues do not allow for the translocation of the bacterial fragments. Most likely, the immune cells remain within the enteric area and degrade the bacteria on site. Some probiotics can regulate metabolic disease [83] and have been involved in the control of bacterial translocation [18] and mucosal defense [75], [84], [85] through mechanisms not yet demonstrated. The current mechanism implies a restoration of the frequency of intestinal Th17 cells [36].

Altogether, the translocation of bacterial fragments and live bacteria to tissues and through impaired gut permeability is a physiological mechanism, which, when unregulated, would lead to a state of chronic inflammation, insulin resistance, adipose tissue proliferation, and metabolic disease. This paradigm strongly depends upon the intestinal defense, notably from the immune and epithelial cells, which acts as a gatekeeper to regulate the flux of bacterial determinant to alter the host and trigger an appropriate response. This function also implies that bacteria and bacterial fragments could be identified in tissues and could have a role. This defines the “tissue microbiota”.

### 2.1 The tissue microbiota: a paradigm shift from the gut with a regulatory role in metabolic diseases

As mentioned in the introduction, the holobiont hypothesis considers an organism along with its microbiome. An uncovered microbiome is the “tissue microbiome,” which states that live bacteria, along with bacterial fragments, reside in the tissues of the host and could regulate its function. Over the last years, we have set up a procedure which enables, way above contaminants that originate from experimental reagents, the quantification and sequencing of the 16S rDNA from the host tissues [86], [87]. From the blood of patients before the onset of type 2 diabetes [88] or heart failure [89], we quantified and characterized an accumulation of specific 16S rDNA sequences defining signatures specific for the prediction of each disease 3–6 years before their occurrence. This set of data demonstrated that an impaired bacterial translocation could lead to, but was not the consequence of, metabolic disease. Specific sets of gram negative bacteria, such as members from the Burkholderiaceae family, accumulate in the stoma vascular fraction from overweight or obese patients almost proportionally to the body mass index [90]. Importantly, the signatures of the tissue microbiota depend upon the tissue considered since different tissues from the same mouse harbor signatures of bacterial DNA, which are specific [86], [87]. Bacterial signatures in visceral adipose tissue closely resembled those in the fecal and ceacum microbiota, while the 16S rDNA sequences from the heart, liver, muscles, and even the cortex of the brain, were characterized by highly different signatures [86], [87]. The impact of the tissue microbiota, and notably of gram negative bacteria, on preadipocytes was studied, and the LPS from tissue microbiota were shown to trigger CD14-dependent macrophage proliferation and preadipocyte proliferation [42]. In the presence of an excess of energy, the preadipocytes differentiate into adipocytes, which contribute to the development of obesity [42]. Simultaneously, adipose tissue macrophages are triggered and produce cytokines, which induce insulin resistance and type 2 diabetes. A large amount of work is still required to understand the molecular mechanisms by which the tissue microbiota regulates the host function, but this paradigm certainly opens the door for discovery. Furthermore, pharmacological strategies aimed at controlling the impact of the tissue microbiota on the host tissue function should control pathological developments such as insulin resistance, obesity, hepatic steatosis, and fibrosis, to cite a few.

## 3 The intestinal immune system protecting the host from gut and tissue microbiota dysbiosis: candidates for metabolic disease

Certainly, the best way to control the changes in gut microbiota ecology that lead to a deleterious and dysbiotic microbiota is to initiate an intestinal defense. Such a defense would be able to fight the presence of a dominant, non-homeostatic, family of bacteria such as the Proteobacteriaceae, whose frequency in the gut, notably at the mucosal layer, increases in response to a fat-enriched diet. This defense could prevent even commensal bacteria from translocating in large numbers to tissues. The first line of intestinal defense is based on the secretion of antimicrobial peptides (AMP) called defensins by intestinal epithelial cells. Defensins are encoded by multiple, highly homologous genes, notably in the jejunum and ileum, in response to gut microbiota colonization throughout life [91]. In response to pathogens or to commensals within the mucosal layer, which are not tolerated by the host immune system, the epithelial cells secrete antimicrobial peptides (AMP). The epithelial cells, Paneth cells, recognize microbial associated molecular patterns (MAMPs) via receptors for MAMPs, TLRs 2,4,5,9, and NLRs and other RIG molecules, to cite a few. The epithelial cells secrete defensins such as alpha, beta-defensin 2,5, and lysozyme [91], [92], which are reduced in obese, human patients [93]. The intracellular TLR-MyD88 pathway is involved and can be triggered by probiotics such as lactobacilli following antibiotic treatment in mice [92] to restore a certain level of defense. Type 2 diabetic patients are characterized by a change in the expression of some AMP in the digestive tract, including the periodontal tissue [94] and the intestine, through mechanisms involving My88 expression in intestinal epithelial cells [36], [95]. In obese individuals, a shift of the active Paneth cell α-defensins, notably the human α-defensin 5 [93], has been observed. Severely obese subjects showed decreased protein levels of both HD5 and lysozyme, while Paneth cell numbers were unchanged; lysozyme protein levels correlated inversely with BMI.

A second line of defense consists of the production of IgAs which can cross the epithelial barrier from the host to the microbiota so that each bacterium is fully covered by immunoglobulin, probably preventing bacterial translocation [96]. The production of IgAs at birth is the consequence of the production by gut microbial factors of ligands of the Ahr (Aryl hydrocarbon receptor) such as indols [97]. The corresponding KO mice have fewer secreted immunoglobulins, and their intestinal barrier is impaired. The IgAs can be provided by the breast milk [98] and protect the host until the intestinal defenses are properly set up. Similarly, the production of IgAs protecting against gut microbiota dysbiosis induced by a high-fat diet can be improved by polyphenols like resveratrol [99], which improves the gut barrier. The immunoglobulins bound at the surface of the bacteria are also an excellent signal for the intestinal immune cell to recognize the bacteria and activate the phagocytosis.

A third line of defense is represented by innate and adaptive immune cells and has been discussed elsewhere [100] (Figure 2). New findings will be discussed here. Metabolic diseases are associated with cellular changes in the innate immune compartment of the intestine [39]. The macrophages, dendritic cells, and poly-nuclear neutrophils are rapid responders, which can capture non-specifically bacterial fragments and bacteria, which have translocated from the mucosal layer to the *lamina propria* or within the Peyer Patches. No dramatic changes in the frequency of the macrophages, and dendritic cells in the intestine have been reported [36]. A change in their function, such as co-activation, is suspected. A subpopulation of dendritic cells expressing the chemokine receptor CX3CR1, activated by frackalin, form transepithelial dendrites enabling the cells to directly sample luminal antigens, can populate the *lamina propria* of the intestine [101]. These cells could be of major importance since they favor the translocation of bacteria from the mucosal layer to the *lamina propria* of the intestine via a MyD88-dependent mechanism [102]. Importantly, depletion of the MyD88 gene, specifically within the epithelium, controls obesity [95], suggesting that such signaling pathway could be important in both the dendritic cells and the epithelial cells for the control of metabolic disease.

In addition, innate lymphoid cells (ILCs) were first described as playing an important role in the development of lymphoid tissues and, more recently, in the initiation of inflammation at barrier surfaces, notably the intestinal epithelial fence, in response to infection or tissue damage [103]. The innate lymphoid cells are immune cells, which do not express specific TCRs directed against a precise antigen neither develop a clonal selection and expansion when stimulated [104] (Figure 2). ILCs represent a first line of lymphoid defense since they promptly respond to bacterial aggressions and tissue injuries by activating the production of AMP and the overall local immune response [105]. This mechanism is linked to the production of large numbers of cytokines such as IL-6, IL-17, IL-22, granulocyte-macrophage colony-stimulating factor, and tumor necrosis factor. Among them IL22 clearly triggers the production of AMP and, therefore, is a direct regulator of non-specific intestinal defense [21] such as during metabolic disease. The nuclear hormone receptor retinoid-related orphan receptor gamma-t (RORγτ) induces a pro-inflammatory program in lymphoid cells leading to the differentiation into ILC1, 2, or 3. ILC3s are very abundant within the intestine and secure the response to bacteria, such as against commensals adhering to the mucosal layer of the intestinal epithelial cells then translocating through the gut to the *lamina propria* during the development of metabolic disease (Figure 2). ILC2s respond to parasites. Notably, ILC3 secretes IL22 and IL17, which could target epithelial cells and enhance the secretion of antimicrobial peptides (AMP), provide non-specific protection against commensals. In addition, a function of ILCs is to regulate the adaptive immune CD4-T cells as shown by genetic or antibody-mediated depletion strategies to target murine ILCs in the presence of an adaptive immune system [104]. ILCs express major histocompatibility complex class II (MHCII) and can process and present the antigen [106], but they limit the commensal bacteria-specific CD4T-cell responses rather than induce T-cell proliferation, which blunts intestinal inflammation. This important study demonstrates that ILCs maintain intestinal homeostasis through MHCII-dependent interactions with CD4T cells that limit pathological adaptive immune cell responses to commensal bacteria. Therefore, an impaired intestinal homeostasis and, hence defense, could be at the origin of metabolic disease. This hypothesis is supported by recent data, which show that obese rodents are characterized by reduced IL-22, resulting in reduced innate lymphoid cells [21]. This seems causal to the disease since IL22 receptor KO mice are characterized by increased body weight gain, although the IL22 KO mice show no metabolic phenotype. However, chronic IL22 treatment improved the metabolic phenotype and various immunological traits in the intestine [21]. More studies identified reduced IL22 production in response to the HFD and metabolic endotoxemia [107].

Another function of ILCs is to regulate the TRegs lymphocytes, which are anti-inflammatory through the secretion of IL10 [108]. Given that intestinal Tregs are under the control of GM-CSF [109], ILCs are the primary source of GM-CSF in the gut [110], and since ILC-driven GM-CSF production is dependent on the ability of macrophages to sense microbial signals and produce interleukin-1beta [111], it was suggested that commensal microbes promote a crosstalk between innate myeloid and lymphoid cells that leads to immune homeostasis in the intestine [110], [111]. Since mice deficient GM-CSF production have altered mononuclear phagocyte effector functions and reduced TReg numbers, their role in the translocation of bacteria to tissue could be implicated. The ILCs can be under the control of dendritic cells (DC) since recent findings support the notion that distinct subsets of classical DCs act on ILCs and T cells similarly to promote either ILC1/Th1/CTL- or ILC3/Th17-type responses [112]. The dendritic cells regulate a first switch in the expression of homing receptors from lymphoid to gut homing receptors, which allows ILC1/3 but not ILC2 to reside in the gut [113]. This function could be only in the presence of the gut-specific tissue factor retinoic acid (RA) [113].

Hence, a hierarchical set of responses to a change of commensal mucosal bacterial would involve the recognition of MAMPs by epithelial cells and phagocytes, i.e. dendritic cells, which could regulate ILCs that, in turn, activate T lymphocytes and reinforce the AMP secretion by epithelial cells [112]. Along the same line, other innate lymphoid cells considered as Mucosa Associated Invariant T cells (MAIT) are mainly present in epithelium such as the intestine [114]. They are also involved in the rapid response to microbiota [115], generating inflammation by means of cytokine production, notably by when recognizing microbiota derived molecules such as riboflavin derivatives. Their non-polymorphic class I receptor MR1 ensures this function. It is important to mention that since only a few studies have been performed on these cells, more data are needed. So far, obese patients are characterized by reduced frequency of circulating MAITs [116], [117]. MAIT cells were more abundant in adipose tissue than in the blood from obese patients and exhibited a striking IL-17 profile, which was partially reversed by bariatric surgery. Therefore, more studies are required to identify the frequency and function of MAIT cells within the intestine.

A large body of evidence demonstrates the major role of T lymphocytes in the control of intestinal defense against commensal microbiota (Figure 2). In the intestine, the γδ-T lymphocytes are the most predominant T cells and their frequency increases in the colon during metabolic disease changes [107]. Their repertoire is restricted, however, and they do not respond to MHC stimulation, which brings into question their role in response to the gut microbiota dysbiosis. However, their frequency was unchanged in the small bowel [36] in the mouse and in obese human [118] which is a major, if not the only, site of bacterial translocation observed during HFD-induced metabolic disease [18]. Conversely, the frequency of the αβ-T lymphocytes was found to be dramatically reduced in the *lamina propria* of the ileum of HFD-fed mice after as short as one week of diet treatment, demonstrating that this impairment was the cause, but not the consequence, of the metabolic disease [36]. The causal role of a reduced frequency of Il-17 producing cells was demonstrated in RORγτ KO mice, which spontaneously develop glucose intolerance over time when fed normal chow. The metabolic phenotype was, at least in part, dependent on the lack of Th17 cells since the transfer of splenic cells, which do not contain ILCs, to a naïve mouse induced hyperglycemia [36]. The impaired Th17-dependent hyperglycemia and impaired intestinal defense was causal to the tissue microbiota dysbiosis and the tissue inflammation leading to insulin resistance [36]. This was due to a reduced phagocyte to lymphocyte co-activation. Microarray analyses from CD45/MHCII/CD4 positive cells and CD3/TCR/CD4 positive cells showed a reduced expression of genes involved in co-activation. In addition, other studies showed that HFD reduced the frequency of Treg in the colon [107]. Importantly, a reduced frequency of the proinflammatory Th1 cells was observed after only 3 weeks, but this was transient as the frequency normalized after 12 weeks of HFD [107]. It is noteworthy that in this study the frequency of Th17 cells in the small bowel and the colon was not changed by the HFD. Additional studies are required to characterize precisely the impact of HFD on the T lymphocyte population of the intestine. Differences in the impact of HFD on gut microbiota could be responsible for such discrepancies. The importance of gut microbiota dysbiosis on the impaired intestinal defense during HFD was demonstrated by fecal microbiota colonization of germ free mice. Two weeks following colonization, recipient mice developed glucose intolerance and a reduced frequency of Th17 cells in the *lamina propria*. The main signature of the dysbiotic gut microbiota induced by the HFD was linked to the reduced frequency of Porphyromonadaceae, which is known to induce Th17. The chronic treatment of HFD-fed mice with a symbiotic tissue microbiota, i.e. a mix between a Bifidobacterium *animalis lactis* 420 and a polydextrose, modified gut microbiota dysbiosis and restored intestinal defense and the control of bacterial translocation restoring a eubiotic tissue microbiota. The eubiotic gut microbiota from the synbiotic-treated HFD-fed mice transferred to germ free mice increased the frequency of IL-17 producing cells and glycemic control. Altogether, the gut microbiota dysbiosis was responsible for the early onset impairment of macrophage to lymphocyte co-activation.

The molecular mechanisms from the gut microbiota responsible for the impaired co-activation could have an epithelium origin. Signals such as frackalin could be impaired and reduce the antigen presenting cell co-activation capacity. Frackalin gene receptor CX3CR1 variants are associated with diabetes [119] as well as metabolic related disorders such as atherosclerosis [120]. In rodents, CX3CR1 deficiency was associated with improved glucose tolerance and insulin sensitivity from hyperinsulinemic-euglycemic clamp [121]. Other hypotheses involve the deleterious impact of bacterial metabolites on the energy metabolism of the antigen presenting cells. To shed some light on these hypotheses, we set up a vaccine strategy using bacterial extracts from the mucosa of the ileum of HFD-diabetic mice [122]. Naïve healthy mice were injected subcutaneously with the bacterial extract from the diabetic mice to trigger and educate the adaptive immune system. Forty-five days later, the mice were fed a high-fat diet to induce hyperglycemia. In such conditions, the vaccinated mice were protected from the development of hyperglycemia [122]. The transfer of splenocytes from vaccinated mice to naïve mice conferred resistance to the high-fat diet-induced metabolic disease demonstrating the role of the adaptive immune system. Interestingly, the gut microbiota dysbiosis was controlled by the vaccine strategy, which increases the concentration of circulating and fecal Immunoglobulins.

## 4 Conclusion

The metabolic disease is at the dawn of new knowledge. Anyone considering studying metabolic disease should take a deep look at gut microbiota diversity and immune responses. Therapeutic strategies, either pharmacological or nutritional, will most likely emerge over the course of the next decade or so. Since it is now clearly demonstrated that the adaptive immune system is engaged in combat against gut microbiota dysbiosis, one could envision setting up a specific vaccine strategy to ensure an efficient intestinal defense against the dysbiotic microbiota. Such strategies should include the notion of biomarkers from gut microbiota dysbiosis, which could classify the patients according to their disease and the efficacy of drugs.

## Conflict of interest

None declared.
